# Supplementary material for: Vesicular-Bound HLA-G as a Predictive Marker for Disease Progression in Epithelial Ovarian Cancer
Source: Cancers (Basel). 2019 Aug 2;11(8):1106. doi: 10.3390/cancers11081106 (PMC6721594; doi:10.3390/cancers11081106)
Supplement: Supplementary file 1 [file cancers-11-01106-s001.pdf]

Supplementary Materials

Vesicular-Bound HLA-G as a Predictive Marker for Disease Progression in Epithelial Ovarian Cancer

Esther Schwich, Vera Rebmann, Peter A. Horn, Alexander A. Celik, Christina Bade-Döding, Rainer Kimmig, Sabine Kasimir-Bauer, and Paul Buderath

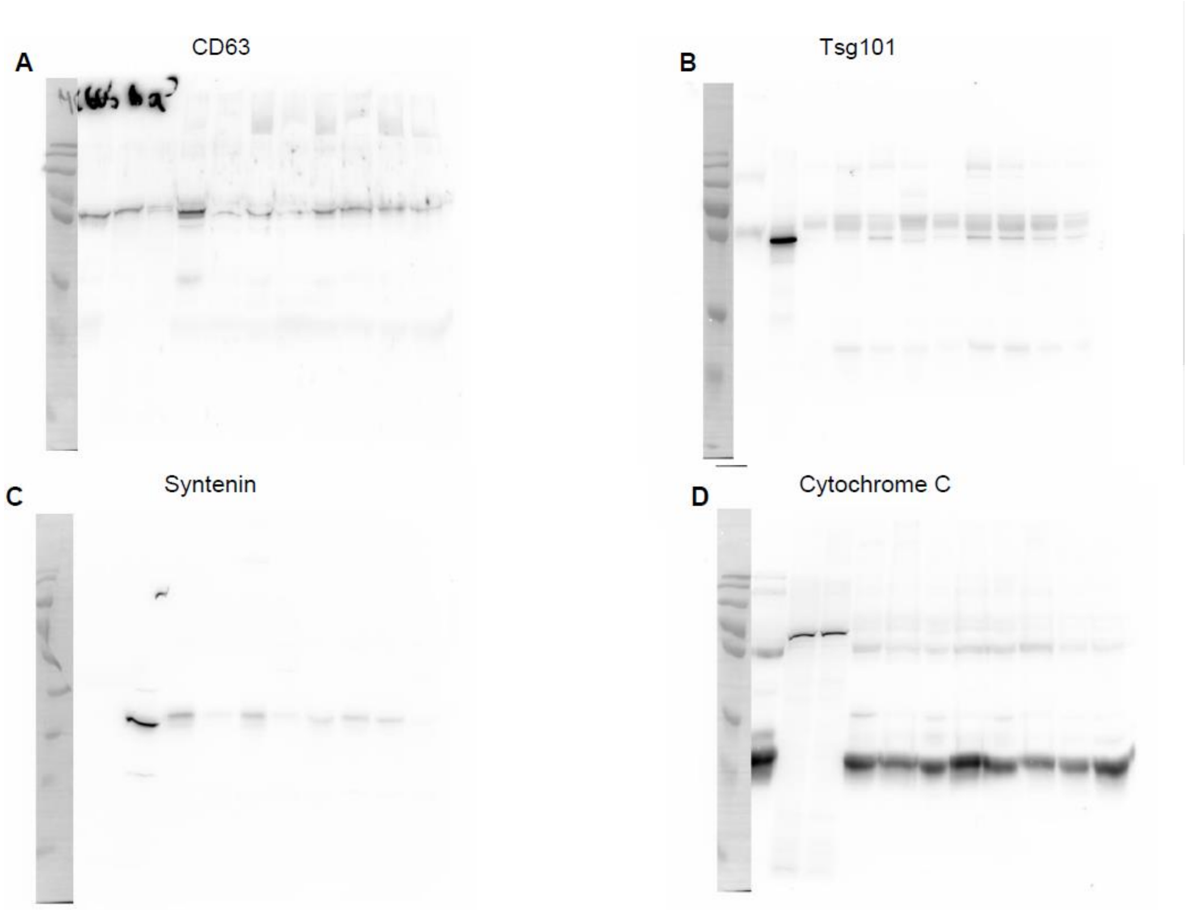

Figure S1. Characterization of EV preparations by western blot.

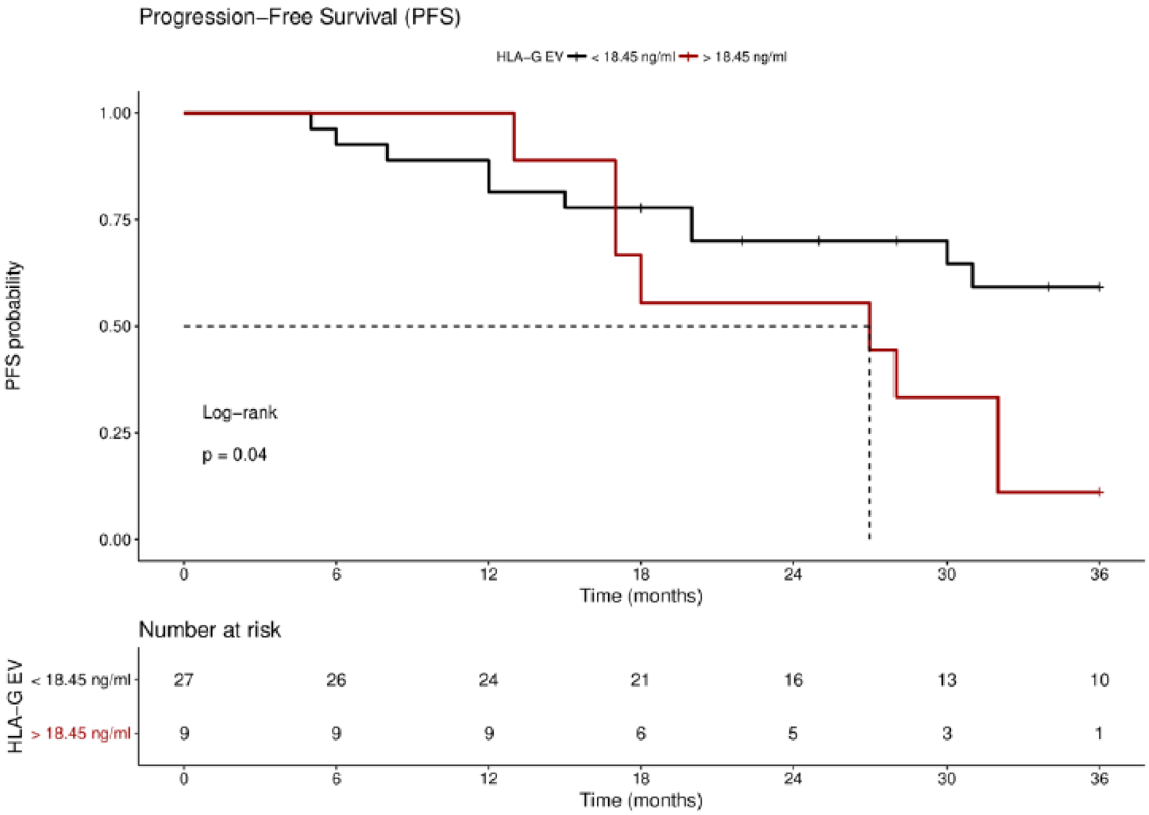

Figure S2. 3-year PFS Kaplan-Meier survival analysis regarding HLA-GEV status of patients without residual tumor burden.

© 2019 by the authors. Licensee MDPI, Basel, Switzerland. This article is an open access article distributed under the terms and conditions of the Creative Commons Attribution (CC BY) license (http://creativecommons.org/licenses/by/4.0/).
